# Cancer of the Breast in Males

**Published:** 1912-10

**Authors:** 


Cancer of the Breast in Males. Pourtau, *Jour. de Med. de Bordeaux,* Feb. 4, 1912. The affection is rare and assumes three forms: cancroid, ulcerated tumor, beginning in skin, secondarily glandular; epithelioma or carcinoma primarily glandular, later involving the skin; sarcoma, developing more slowly and with better prognosis than in women

